# Visceral leishmaniasis lethality in Brazil: an exploratory analysis of associated demographic and socioeconomic factors

**DOI:** 10.1590/0037-8682-0007-2020

**Published:** 2020-09-11

**Authors:** Lucas Edel Donato, Lúcia Rolim Santana de Freitas, Elisabeth Carmen Duarte, Gustavo Adolfo Sierra Romero

**Affiliations:** 1 Ministério da Saúde, Secretaria de Vigilância em Saúde, Brasília, DF, Brasil.; 2 Universidade de Brasília, Faculdade de Medicina, Programa de Pós-Graduação em Medicina Tropical, Brasília, DF, Brasil.

**Keywords:** Visceral leishmaniasis, Acquired Immune Deficiency Syndrome, Mortality

## Abstract

**INTRODUCTION:**

It is believed that delays in diagnosis and treatment of Visceral Leishmaniasis (VL) contribute significantly to the burden of VL lethality in Brazil.

**METHODS:**

This study included several parts: a descriptive cross-sectional study of the individual characteristics of deaths from disease; a descriptive ecological study of the spatial distribution of deaths from disease; and an ecological analytical study to evaluate the association between disease lethality rates and the demographic, socioeconomic, and health indicators. The study population comprised all cases diagnosed throughout the country per the National Disease Notification System (SINAN) and the total number of disease deaths recorded in the Mortality Information System (SIM) from 2007 to 2012.

**RESULTS:**

Of the 223 deaths from disease captured by pairing the databases, 59.1% were reported as "death from other causes". There were significant associations between VL lethality rate and municipalities with the highest proportion of vulnerable individuals (rate ratio (RR)=1.18, 95% confidence interval (CI): 1.01-1.27), with VL lower incidence rate (RR=0.62, 95% CI: 0.58-0.67) and a higher incidence rate of Acquired Immune Deficiency Syndrome (AIDS) (RR=1.20, 95% CI: 1.17-1.51).

**CONCLUSIONS:**

Linking the SINAN and SIM databases allowed the inclusion of 14% of otherwise underreported deaths from VL for the study period, showing that this method is useful for the surveillance of VL-related deaths. The size of the municipal population, proportion of the vulnerable population, incidence of disease, and the incidence of AIDS were associated with municipal lethality rates related to VL in Brazil.

## INTRODUCTION

Visceral leishmaniasis (VL) is an infectious disease caused by protozoans of the genus *Leishmania.* In Brazil, VL is associated with infection by *Leishmania infantum* (syn. *L. chagasi*) infections, and presents through a zoonotic cycle in which the domestic dog constitutes the main reservoir of the parasite^1^. The vectors responsible for transmission of the disease in the country are *Lutzomyia longipalpis* and *Lutzomyia cruzi* ^2^. About 90% of new cases are estimated to be concentrated in seven countries, and in 2017 a total of 20,792 cases were reported in these countries^3^. In the Americas, most cases of VL occur in 12 Latin American countries, and 90% of these occur in Brazil^4^.

The Brazilian Ministry of Health has identified reduction of VL lethality as the goal of the Visceral Leishmaniasis Surveillance and Control Program (Programa de Vigilância e Controle da Leishmaniose Visceral (PVC-VL))^5^. The guidelines described to achieve this goal include early diagnosis and treatment, serological surveys in dogs, vector control, and health education^6^. In fact, it is believed that late diagnosis and treatment of the disease contribute significantly to the burden of VL lethality in Brazil^7^.

In recent years, there has been some progress in the search for solutions to combat VL, especially with regard to diagnosis and treatment. However, few studies have addressed issues related to prognosis, with disease lethality as the outcome^8^.

The fatality rate for VL in Brazil has increased gradually in recent years, from 3.6% of diagnosed cases in 1994 to 8.5% in 2003. This rate decreased between 2003 and 2007, but starting from 2008, the lethality rate again increased to reach 7.1% in 2012^9^. In Campo Grande (the capital of the state of Mato Grosso do Sul), where diagnostic tracking of human cases of VL began in 2001, the mortality rate reached 7% with a specific mortality rate of 9.1 per 100,000 inhabitants. These statistics demonstrate the severity of the problem^10^. A more critical situation was observed in the capital city of Belo Horizonte, which experienced a lethality rate of 23.6% in 2009, surpassing the national average of the same year^11^.

In addition, VL lethality observed in people living with Human Immunodeficiency Virus (HIV)/Acquired Immune Deficiency Syndrome (AIDS) (PLHA) has been receiving increasing attention^12^. The estimated lethality rate of VL/AIDS co-infection in Brazil between 2001 and 2010 was approximately 25%, which corresponds to three times the lethality rate observed in non-coinfected individuals^13^.

Because it is a neglected disease which occurs mainly in tropical countries, the burden of VL falls disproportionately on the poorest segments of the global population. Lack of access to healthcare is associated with late diagnosis and treatment, and with increased VL morbidity and mortality^14^. Public investment in care activities, combined with the most effective surveillance tools, could contribute to reducing the burden and magnitude of VL^4^.

In view of the above, it is believed that increasing knowledge of the factors associated with VL lethality can help improve the currently recommended public health policies, making it possible to increase the number of epidemiological surveillance programs in municipalities and states, thereby avoiding VL-related deaths. Therefore, the objective of this study was to identify socioeconomic, demographic, and health factors of the municipalities associated with lethality due to VL in Brazil from 2007 to 2012.

## METHODS

### Study design

The study was divided into three stages: a descriptive cross-sectional study of the individual characteristics of deaths from VL; a descriptive ecological study of the spatial distribution of deaths from VL; and an ecological analytical study to evaluate the association between VL lethality rates and the demographic, socioeconomic, and health indicators of Brazilian municipalities.

### Study scope and population

The study population comprised all VL cases diagnosed throughout the country per the National Disease Notification System (SINAN) and the total number of VL deaths recorded in the Mortality Information System (SIM) from 2007 to 2012. For the ecological approach, all 5,569 Brazilian municipalities were included.

### Definition of incident case and death from VL

A case of VL was considered to be any case recorded in SINAN for which the variable "final classification" was classified as "confirmed."

### Deaths were identified in two ways:

1) cases of VL recorded in SINAN for which the variable “progression” was classified as "death from VL"; and

2) cases of VL recorded in SINAN (without information on progression to death) paired with notification in the SIM during the same period, with the underlying cause of death in the latter system being VL, bleeding, sepsis, or HIV. For the HIV variable we chose the ecological approach considering that the status of this variable was not available for a relevant number of cases in the SINAN database.

To link the SINAN and SIM databases for the identification of VL deaths not recorded in SINAN, probabilistic pairing was applied using Python Brasil with a bloom filter^15^. This program automatically identifies records while considering parameters which are probabilistically estimated. The following variables were used in the comparison fields: patient name, mother's name, and date of birth.

After identifying the records, the notifications which could not be submitted to the pairing process between the banks because they exhibited some inconsistencies among the comparison variables were evaluated by three observers. For these cases, the following pre-established pairing criteria were used: date of being reported into SINAN; date of birth of the individual and the mother; date of death in the SINAN and SIM databases; and municipality of residence. After this evaluation, cases with consistency in the variable data defined above were considered to be deaths.

### Spatial distribution of deaths from VL

For the spatial distribution of deaths from VL, the municipality of residence identified in the notification records was considered as an aggregated level of the spatial units. Thematic maps were created using the TerraView 4.2.2 software, and the municipal layers were obtained from the censuses of the Brazilian Institute of Geography and Statistics (IBGE).

### Socioeconomic, demographic, and health variables of municipalities

The variables described in Box 1 were explored. Socioeconomic variables were obtained from the Atlas of Human Development in Brazil, published in 2013 by the United Nations Development Program^16^. This atlas sources data and indicators from the last three demographic censuses conducted by the Brazilian Institute of Geography and Statistics^17^. Population data were obtained from the Brazilian Institute of Geography and Statistics^17^.

Healthcare-related variables were obtained from the National Registry of Health Establishments (CNES) and the Department of Primary Health Care (DAB) of the Secretariat of Health Care (SAS), Ministry of Health. Disease-related variables were obtained from SINAN and the Information Technology Department of the Unified Health System^18^.

### Statistical analysis

In the descriptive step of data analysis, the absolute and relative frequencies of the variables characterizing the cases of and deaths from VL in the evaluated period were calculated. Maps were created for the analysis of the spatial distribution of cases and deaths from VL, and the municipality of residence of the notification records was used for spatial unit aggregation.

In the analytical stage, the ecological associations between the characteristics of the Brazilian municipalities and the VL lethality rate were evaluated. For this purpose, the individual effects of categorical and continuous independent variables associated with the lethality rate were evaluated by a zero-inflated Poisson model with a link function log (λ)^19^ ^,^ ^20^, using the statistical software Stata® 11.2. The estimates adjusted using the zero-inflated Poisson model were obtained as follows. A Pearson correlation matrix was first developed to identify collinearity between the independent variables of interest. Variables with correlation coefficients r≥0.8 were considered collinear, and only one of the variables was selected for the model. This choice was based on parameters such as the ease of measurement of the variable and its potential for being understood by decision-makers. Next, some continuous variables were categorized based on the behavior observed in the preliminary bivariate analysis. 

Finally, multivariate models were constructed. Variables exhibiting a statistically significant association with the lethality rate, defined by a p-value < 0.20, were included. Multivariate analysis was conducted using a hierarchical approach with the model incorporating two levels: the distal, including demographic variables; and the proximal, including health indicators. The backward method was used on each of the hierarchical levels to select the independent variables.

Distal variables were added first to the multivariable model, and retained as long as they remained significantly (P<0.05).

### Ethical aspects

The project safeguarded the privacy of the included participants by maintaining confidentiality of patient names in VL cases reported in the SIM and SINAN databases. All ethical precepts of human research were implemented. The project was approved by the Research Ethics Committee of the School of Health Sciences of the University of Brasília (CEP/FS-UnB) on March 13, 2014 (CAAE n. 22229613.5.0000.0030). The research was conducted in accordance with the principles of Declaration of Helsinki.

## RESULTS

In the period from 2007 to 2012, 1,368 deaths from VL in Brazil were notified in SINAN. After pairing the SINAN and SIM databases, 223 additional deaths were identified which were not indicated as “death from VL” cases in the “progression” field of the SINAN notification form. Thus, the total number of deaths identified for the period studied was 1,591. In 2010, the highest number of deaths from VL (n=48, 21.5%) was recorded, captured by pairing the SINAN and SIM databases (Table 1). Of the 223 deaths from VL captured by pairing the databases, 19.7% (44/223) had "cure" recorded in the “progression” field of the SINAN form; 59.1% (132/223) had "death from other causes" recorded; 14.7% (33/223) reported "not [being] informed"; and 6.2% reported "transferred" (14/223).

**TABLE 1:** Distribution of visceral leishmaniasis deaths identified in the National Disease Notification System (SINAN) and through the linkage of this database with the Mortality Information System (SIM) - Brazil, 2007 to 2012.

Year of notification	Deaths from VL from	Additional Deaths from VL identified through
	SINAN database	database linkage
2007	191	38
2008	220	33
2009	228	36
2010	230	48
2011	262	30
2012	216	38
Total	1.368	223

Source: **SINAN:** Sistema de Informação de Agravos de Notificação/Secretaria de Vigilância em Saúde/Ministério da Saúde; **SIM:** Sistema de Informações sobre Mortalidade.

**FIGURE 1:**
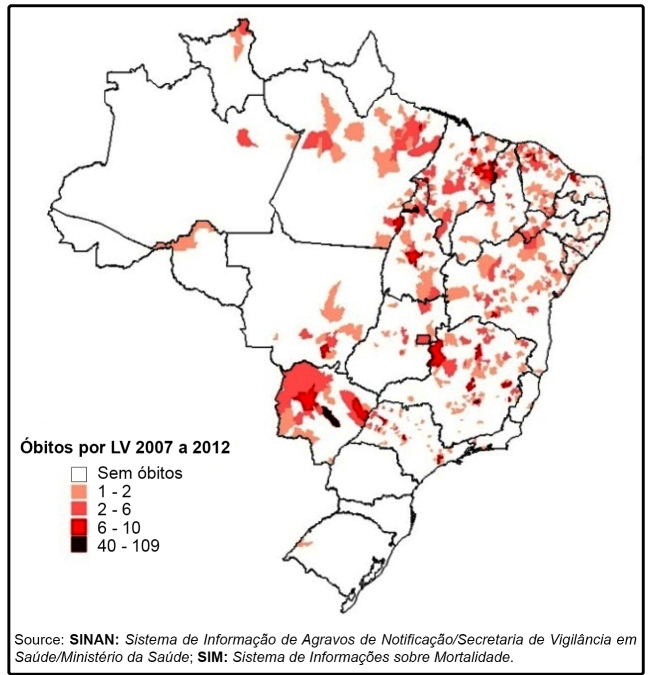
Distribution of deaths from Visceral Leishmaniasis in 2007 to 2012.

Figure 1 shows the distribution of total deaths from VL (n=1,591) identified in Brazil between 2007 and 2012. It is possible to observe a concentration of deaths from VL in the Northeast region, though there is a distribution of VL-related deaths throughout the entire country (Figure 1).

Deaths from VL in Brazil predominantly affected men (66.8%) during the period from 2007 to 2012. Analysis by age group in the same period showed a predominance of adults aged 35 to 49 years, representing 21% of deaths (Table 2). The accumulated deaths in this period were 1,591, with an annual average of 260 deaths; the year 2007 had the lowest recorded number of deaths (n=234), whereas 2011 showed the highest number of recorded deaths (n=284).

The variables selected to evaluate association with the VL lethality rate in Brazilian municipalities during the 2007-2012 period are described in Table 3.

**TABLE 2:** Proportional distribution of deaths from visceral leishmaniasis according to age group. Brazil, 2007-2012.

Age group	Deaths from visceral leishmaniasis	
	Number	%
<1 year	187	12.0
1-4	180	11.5
5-9	40	2.6
10-14	35	2.2
15-19	52	3.3
20-34	243	15.6
35-49	329	21.1
50-64	260	16.7
65-79	169	10.8
>80	64	4.1
Total	1559	100

Source: **SINAN:** Sistema de Informação de Agravos de Notificação/Secretaria de Vigilância em Saúde/Ministério da Saúde; **SIM:** Sistema de Informações sobre Mortalidade.

**TABLE 3:** Description of the variables selected to evaluate association with the lethality rate from visceral leishmaniasis in Brazilian municipalities during the 2007-2012 perQiod.

Variables	Description
**Socioeconomic**	Proportion of population in extreme poverty
	Municipal Human Development Index (MHDI)
**Demographic**	Age; sex
	Proportion of vulnerable population
	Municipal population size
**Healthcare-related**	Coverage by the Family Health Strategy
	Hospital beds per inhabitants
	Doctors per inhabitants
**Disease-related**	Visceral leishmaniasis incidence rate AIDS incidence rate

**AIDS:** Acquired Immune Deficiency Syndrome.

In the crude (bivariate) analysis, the VL lethality rate was significantly associated with small municipalities (municipal population >20,000 and <100,000 inhabitants (p<0.001)) including a higher proportion of vulnerable population (p<0.001), with a lower incidence rate of VL per 100,000 inhabitants (p<0.001), and with a higher incidence of AIDS per 100,000 inhabitants (p<0.001). In the adjusted analysis, small municipalities (population 20,000 to <50,000) were associated with higher rates of VL lethality (rate ratio (RR)=1.90; 95%CI: 1.68-2.15 for population size 20,000 to <50,000 and RR=1.43, 95%CI: 1.29-1.60 for population size 50,000 to <100,000) compared to municipalities with a population of <20,000 inhabitants. There were also significant associations between the VL lethality rate and municipalities with the highest proportion of vulnerable individuals (RR=1.18, 95%CI: 1.01-1.27), with a lower incidence rate of VL (RR=0.62, 95%CI: 0.58-0.67), and a higher incidence rate of AIDS per 100,000 inhabitants (RR=1.20, 95% CI: 1.17-1.51) (Table 4).

**TABLE 4:** Crude and adjusted analysis of factors associated with the rate of lethality due to visceral leishmaniasis in Brazil, 2007 to 2012.

	Crude analysis		Adjusted analysis a	
Characteristics	RR^b^	p-valor	RR^b^	p-valor
	(IC 95%)		(IC 95%)	
**Demographic**				
Population size				
(Reference <20,000 inhab.)				
20,000 to <50,000 inhab.	1,28	0,000	1,90	0,00
	(1,16 a 1,41)		(1,68 a 2,15)	
50,000 to <100,000 inhab	1,12	0,017	1,43	0,00
	(1,02 a 1,23)		(1,29 a 1,60)	
≥100,000 inhab.	0,98	0,671	1,09	0,14
	0,88 a 1,09		(0,97 a 1,23)	
Vulnerable population (%)^c^	1,03	0,000	1,18	0,00
	(1,02 a 1,04)		(1,01 a 1,27)	
**Socioeconomic**				
Gini Index (0 to 1)	1,28	0,398	-	-
	(0,71 a 2,31)			
MHDI^d^	1,31	0,229	-	-
	(0,84 a 2,03)			
Population in extreme poverty (%)	1,09	0,069	-	-
	(0,99 a 1,07)			
**Health care-related**				
Hospital beds/inhab.	1,02	0,479	-	-
	0,97 a 1,01			
Doctors/inhab.	1,01	0,552	-	-
	(0,97 a 1,01)			
Coverage of the Family Health	1,00	0,065	-	-
Strategy (%)	(0,99 a 1,22)			
**Disease-related**				
Incidence rate of visceral leishmaniasis (per 100,000 inhabitants)	0,74	0,000	0,62	0,00
	(0,70 a 0,79)		(0,58 a 0,67)	
Incidence of AIDS (per 100,000 inhabitants)	1,07	0,015	1,20	0,00
	(1,00 a 1,83)		(1,17 a 1,51)	

a Zero-inflated Poisson model (ZIP); ^b^Rate ratio; ^c^Population of individuals younger than 1 and older than 50 years of age of a given municipality; ^d^Municipal Human Development Index.

## DISCUSSION

Reducing the VL lethality rate is currently the main goal of the control program designed by the Brazilian Ministry of Health^21^. The lethality of VL can be explained by a range of factors, such as late diagnosis and treatment, toxicity of drugs available for treatment, comorbidities, poor healthcare quality, and socioeconomic factors related to the individual and the environment^22^. Timely and accurate diagnosis remains a challenge in Brazil, as in other affected countries, where the disease is still treated only on the basis of clinical suspicion. The use of tests with good accuracy which are easy to perform, such as rapid tests employing recombinant proteins, has opened a space for improving the decision-making process for health professionals, regarding the provision of timely treatment^23^ ^,^ ^1^ ^,^ ^24^.

The results of the present study point to a higher lethality rate in small municipalities (20,000 to fewer than 100,000 inhabitants). This result could be explained in part by the instability of lethality rates in municipalities of this size, where few cases are diagnosed. In the literature, it is possible to observe an association between lethality and larger municipalities which have a more complex healthcare structure and attract more severe cases, thus selecting for individuals with higher risk of death^25^. In these cases, the place of death and not the place of residence of the patient is considered in the analysis/notification of the event. This phenomenon could explain the lower lethality rate in municipalities with fewer than 20,000 inhabitants relative to municipalities with 20,000 to 100,000 inhabitants, which would most likely select for patients with less severe VL. However, the trend in lethality rates in municipalities with more than 100,000 inhabitants still remains to be explained. Because the disease generally has a subacute or chronic course, even considering that the quality of and access to healthcare is precarious in municipalities with fewer than 20,000 inhabitants, there would be an opportunity to seek care in larger and denser neighboring municipalities. This may improve the lethality rate of the smaller municipalities because the deaths would then be recorded in the municipality where they actually occurred and not in the municipality where the patient resided. Other factors may include migration of rural populations to the periphery of large urban centers with more than 100,000 inhabitants, coupled with poor housing, uncontrolled deforestation, and a high number of infected dogs, which would contribute to the increase in VL cases and consequently to death from the disease^26^. In contrast, the spread of the zoonosis to areas with a large population density could negatively affect lethality, as a result of the lack of knowledge about the disease, both among the most vulnerable population and among the healthcare teams responsible for providing services in these places. In this scenario, the development of a healthcare network with qualified referral centers which can quickly accumulate the experience necessary for proper management of the most serious cases and associated complications, becomes essential.

The observed historical trend suggests the existence of a population vulnerable to the disease in the age groups corresponding to the extremes of life: individuals younger than 1 year old and those older than 50 years. In the present study, it was observed that on average, one-fifth of the population studied in this period met this profile, and an association of lethality with the proportion of vulnerable population was found. Factors potentially related to this greater vulnerability of individuals in these age groups may be the time between the onset of first symptoms and physician consultation, malnutrition, immune problems, and the presence of other associated pathologies which may play an important role in the genesis of an unfavorable outcome^9^ ^,^ ^27^ ^,^ ^28^ ^,^ ^29^.

The ratio of doctors to number of inhabitants and coverage by the family healthcare plan was not significantly associated with VL lethality in this study. In a study which evaluated the determinants of the causes of mortality, a negative association between infant mortality and the number of doctors was identified^30^. It is important to note that in the present analysis, all medical specialties were included, and a large proportion of these professionals do not participate directly in the diagnosis and treatment of VL, which may explain the observed result.

Extreme poverty was not significantly associated with VL in bivariate analysis; however, some studies have found that income may be associated with VL. We found that in 47% of the interviewed cases, the income was below the minimum wage, set at BRL120.00 in 1997. In another study, it was identified that increased income may be associated with a decrease in the occurrence of VL^1^ ^,^ ^31^.

None of the indicators directly related to poverty was associated with VL lethality in the period studied. Given that VL has been classically associated with poverty, the observed result may be explained by the municipalities which present with significant extreme poverty and a low human development index (HDI), combined with a long history of the disease in the community. In this scenario, despite the difficulties faced by the local health services, there would be accumulation of knowledge and skills for the management of patients with VL, including the timely identification of the need for referral of the most severe patients to referral centers with greater technological density, which could reduce disease lethality. As a counterpoint, there would be municipalities with a lower proportion of population in extreme poverty and higher HDI, where the disease was introduced recently; such conditions may worsen the quality of case management due to the inexperience of health teams, resulting in a negative impact on lethality. We identified areas at high risk of VL based on socioeconomic indicators and remote sensing data, and concluded that even with the complexity involved in this approach, it was possible to observe a relationship between disease spread and socioeconomic and environmental factors^32^.

The incidence of AIDS was associated with a lethal outcome. Carvalho and Cols. (2013) found an association between VL/HIV coinfection and death^33^. The epidemiological profiles of AIDS and VL have changed in Brazil; data suggest that the interiorization of HIV infection simultaneously with the urbanization of VL may be responsible for the greater exposure of the population to both infections^34^. It was also reported that the municipalities with the highest number of notifications of VL/HIV coinfection cases were among the 20 municipalities with the highest incidence of AIDS^35^.

In addition to the pathophysiological factors of the disease which may contribute to an unfavorable outcome in co-infected patients, the use of the recommended medications for the treatment of VL may also contribute to death. The deleterious effect of the use of pentavalent antimonials on PLHA is already known. A cohort study reported that HIV infection is an independent predictor of poor outcome at six months after VL diagnosis^36^.

The methodology used in the present study has the limitations intrinsic to ecological studies whose conclusions are limited to the aggregated data and do not allow the establishment of causal relationships at the individual level. Our choice of using the HIV incidence rate instead of the individual HIV status data because of the unavailability of these data in the SINAN records is an example of that limitation. Another relevant limitation refers to the use of secondary data, which may not accurately represent real life conditions. In the present study, the linkage between the databases was not directed toward detecting the underreporting of VL cases but, rather, to correct classification errors of VL cases recorded as survivors when they had actually died of VL. In this sense, the denominator of the number of VL cases was limited to SINAN records, which may have been affected by underreporting. The socioeconomic and demographic variables used were not specifically obtained to try to understand the process of illness and death by VL; therefore, they could have suffered from measurement bias because they were primarily collected for other purposes.

In conclusion, in this study, the linkage of the SINAN and SIM databases allowed for the inclusion of 14% of otherwise underreported deaths from VL for the study period, showing that it is a useful tool for the surveillance of this event. It was found that the size of the municipal population, the proportion of the vulnerable population, the incidence of VL, and the incidence of AIDS were associated with municipal lethality rates from VL in Brazil during the study period. These variables may help healthcare managers intervene to reduce the vulnerability of municipalities with these characteristics, to the fatal outcomes of VL.
